# Topiramate plus nortriptyline in the preventive treatment of migraine: a controlled study for nonresponders

**DOI:** 10.1007/s10194-011-0395-4

**Published:** 2011-10-19

**Authors:** Abouch Valenty Krymchantowski, Carla da Cunha Jevoux, Marcelo E. Bigal

**Affiliations:** 1 Headache Center of Rio, Rua Siqueira Campos 43/1002 Copacabana, Rio de Janeiro, 22031-070 Brazil; 2 Chief Medical Office, Merck inc, Whitehouse Station, USA; 3 Department of Neurology, Albert Einstein College of Medicine, Bronx, NY USA

**Keywords:** Topiramate, Nortriptyline, Migraine, Prevention, Combination, Therapy

## Abstract

A sizeable proportion of migraineurs in need of preventive therapy do not significantly benefit from monotherapy. The objective of the study is to conduct a randomized controlled trial testing whether combination therapy of topiramate and nortriptyline is useful in patients who had less than 50% decrease in headache frequency with the use of the single agents. Patients with episodic migraine were enrolled if they had less than 50% reduction in headache frequency after 8 weeks of using topiramate (TPM) (100 mg/day) or nortriptyline (NTP) (30 mg/day). They were randomized (blinded fashion) to have placebo added to their regimen, or to receive the second medication (combination therapy). Primary endpoint was decrease in number of headache days at 6 weeks, relative to baseline, comparing both groups. Secondary endpoint was proportion of patients with at least 50% reduction in headache frequency at 6 weeks relative to baseline. A total of 38 patients were randomized to receive combination therapy, while 30 continued on monotherapy (with placebo) (six drop outs in the combination group and three for each single drug group). For the primary endpoint, mean and standard deviation (SD) of reduction in headache frequency were 4.6 (1.9) for those in polytherapy, relative to 3.5 (2.3) for those in monotherapy. Differences were significant (*p* < 0.05]. Similarly, 78.3% of patients randomized to receive polytherapy had at least 50% headache reduction, as compared to 37% in monotherapy (*p* < 0.04). Finally we conclude that combination therapy (of TPM and NTP) is effective in patients with incomplete benefit using these agents in monotherapy.

## Introduction

Migraine is a chronic neurological disorder with episodic attacks of headache and associated symptoms [1, 2]. The disability of migraine can be severe and imposes a considerable burden on the sufferer and the society [3–5].

Because migraine resembles both acute and chronic conditions, pharmacological treatment is often divided into acute and preventive modalities [6]. Preventive treatment is recommended for patients with frequent or disabling attacks [5]. Frequently used first-choice medications for the preventive treatment of migraine include beta-blockers, tricyclic antidepressants, calcium channel antagonists, and neuromodulators [7, 8]. When properly used, preventive medications are associated with improvement in the quality of life [9] and decreased disability [10]. Nonetheless, a sizeable proportion of migraineurs in need of preventive therapy do not significantly benefit from monotherapy (i.e. do not experience meaningful reduction in headache frequency) or experience side effects that impact adherence [11].

Clinical experience and limited evidence suggests that combination preventive therapy benefits individuals with poor response to monotherapy, although controlled studies are not available. Accordingly, the objective of this study was to conduct a randomized controlled trial testing whether combination therapy of topiramate and nortriptyline is useful in patients who had less than 50% decrease in headache frequency with the use of the single agents. We hypothesized that, in patients with incomplete migraine relief using monotherapy, polytherapy is associated with improved outcomes as compared to maintaining monotherapy.

## Methods

Our sample consisted of 80 individuals (20–60 years of age) selected from one outpatient headache clinic. All had episodic migraine according to the Second Edition of the International Classification of headache disorder [12] for at least 1 year. Sample was recruited during the years of 2005 and 2006. Of them, 40 were using Topiramate (TPM) 50 mg bid for nearly 6 weeks (labeled titration schedule). Other 40 individuals were using nortriptyline (NTP) 30 mg/day for nearly 6 weeks (titration of 10 mg at nighttime for 7 days, 20 mg for 7 days, and 30 mg thereafter).

To be included in this study, patients had to have less than 50% headache frequency improvement at 8 weeks, relative to baseline, as documented by headache calendars. Patients should also empirically consider that the benefit of preventive medication had not been adequate (meaning they were not satisfied with their level of improvement). Exclusion criteria include women not using stable contraceptive methods for at least 3 months as well as patients with less than 4 or with more than 12 headache days per month. The chosen headache frequency limits as well as the frequency of rescue medications consumption were arbitrary. Additionally, patients with comorbid relevant psychiatric or medical conditions were not included as evaluated by a detailed first time visit of 1 h in addition to Hamilton anxiety and depression scales as well as Beck inventory. Participants were patients consulting a tertiary headache center, who were found to meet inclusion criteria during a routine medical visit. This method was chosen to mimic neurology clinical practice, since providers often face complaints of incomplete migraine relief when patients are using standard medications, and have to base decisions with limited supportive evidence. Trial was therefore conducted in a single headache center, in Rio de Janeiro, Brazil.

After agreeing to participate (documented by signing an Institutional Review Board (IRB) approved consent form), patients originally using TPM in monotherapy were randomized to also receive NTP or placebo at a 1:1 ratio, using random number generation trough a software. They were titrated to two capsules of NTP or matching placebo after 1 week and three capsules during dinner for 6 weeks (30 mg of NTP or matching placebo).

Patients using NTP in monotherapy (30 mg/day) added TPM or placebo as follows: one capsule (25 mg or placebo during breakfast) for 7 days; one capsule of 25 mg TPM or matching placebo bid for 10 days; one capsule in the morning and two at nighttime for 14 days; two capsules bid thereafter. Accordingly, those randomized to receive TPM reached a dose of 100 mg. The design was therefore parallel. After failure of monotherapy (run-in, open label and prior to randomization), patients were randomized to continue in monotherapy (drug + placebo) or to be switched to polytherapy. Reasons to use TPM or NTP as initial therapeutic options were based on the first author’s experience and did not follow any specific characteristic of the patients such as previous failure or use of pharmacological classes.

All patients received emphatic education on the treatment of migraine and received the study drugs for free, which were the commercially available 25-mg capsules of TPM and the 10-mg capsules of NTP. The placebo capsules had the same appearance. Headache frequency and severity was captured using detailed headache calendars, and revised monthly. Rescue medications were allowed and limited to twice a week since it is the maximum allowed frequency of headache medications intake used as routine by the study center. They consisted of a combination of a triptan plus a nonsteroidal anti-inflammatory drug. The study was approved by an Investigational Review Board.

Since this study was developed to mimic conditions often used in clinical practice, we achieved endpoints after 6 weeks of therapeutic dose or around 10 weeks after randomization. Endpoints were defined a priori. Primary endpoint was reduction in the number of headache days at 6 weeks versus baseline, comparing both the groups. Secondary endpoint was proportion of patients with at least 50% reduction in headache frequency, comparing both the groups. At the termination visit (6 weeks) patients were specifically prompted to report any adverse events. Patients completed at least three visits in order to be evaluated (one for initial prescription of the single drug, one for evaluating the headache outcome with the initial chosen drug, when the inclusion of the second drug was carried out and a third visit to evaluate the outcomes with the two drugs).

### Statistical analyses

The study was powered a priori, for the primary endpoint. We assumed a difference of 1.5 days between both groups with a standard deviation (SD) of 1.5, based on prior preventive clinical trial studies. Using 1-sided *T* test we needed 38 patients per group to have a 80% power to detect a difference at the 5% level. Descriptive statistic and summary tables were developed. Normality of data was assessed using the Kolmogorov–Smirnov test. Data necessary for the primary endpoint was found to follow a normal distribution and were compared with the unpaired, 1-sided *T* test. For the secondary endpoint (proportions), data were compared using the Chi-Squared test. Multiple comparisons were not performed, therefore corrections were not necessary. Due to sample size limitations, we did not conduct sub-analyses or estimated response as a function of demographics or migraine features. Significance level was established at 5%.

Since assessment was conducted at 6 weeks only, we conducted per-protocol analyses, since intent-to-treat analyses do not apply (efficacy was assessed at a single point in time).

## Results

### Overall description

Table 1 describes the demographics and baseline frequency of participants in group 1 (TPM), group 2 (NTP) and overall. In group 1, 6 (15%) men and 34 (85%) women were included (ages 22–57, mean 36 years). The mean overall baseline headache frequency (HF) was 7 ± 3 headaches days/month. Among them, 23 (57.5%) received NTP and 17 (42.5%) were randomized to receive placebo. Group two included 9 (22.5%) men and 31 (17.5%) women (ages 20–60, mean 39 years) who had a baseline mean HF of 9 ± 3 headaches days/month. Among them, 21 (52.5%) received TPM and 19 (47.5%) received placebo. None of the patients had comorbid tension-type headache

**Table 1:** Demographic and headache characteristics of participants

	TPM + NTP *N* = 44	TPM + Placebo *N* = 17	NTP + Placebo *N* = 19
Demographics	36 women (81.8%) Ages 36 ± 9.55	15 women (88.2%) Ages 35.7 ± 8.2	16 women (84.2%) Ages 41.5 ± 6.72
	8 men (18.2%) Ages 36.6 ± 9.47	2 men (11.8%) Ages 36–38	3 men (15.8%) Ages 39.6 ± 6.77
Baseline headache days/month	8.1	8	8

* Age presented as mean ± standard deviation

**Fig. 1:**
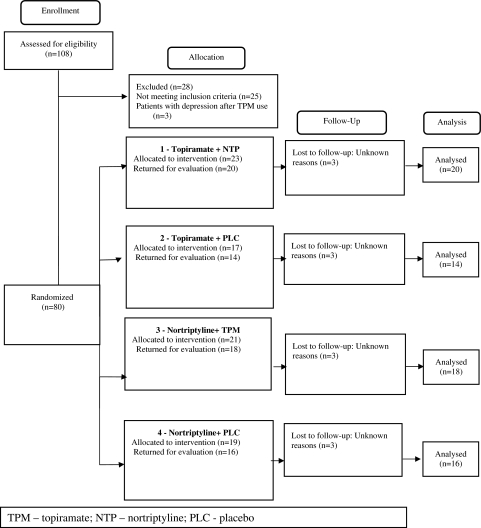
Participants flow diagram

Accordingly, a total of 44 patients (8 men and 36 women) were randomized to receive the combination of TPM and NTP (23 received NTP and 21 received TPM). Seventeen patients (2 men and 15 women) took TPM and placebo and 19 (3 men and 16 women) had NPT and placebo (Fig. 1).

### Headache parameters

#### Reduction in headache frequency

Unless otherwise stated, we present means and SD of the data. Mean reduction in headache frequency at follow-up relative to baseline was significantly higher in the combination group versus monotherapy [mean 4.6 (SD 1.9) versus 3.5 (SD 2.3), *p* = 0.04]. When analyzing by subgroup, headache frequency significantly dropped in all groups after randomization. In patients with previous incomplete relieve to TPM that were randomized to have NTP added, mean monthly headache frequency significantly dropped from a mean of 8.1 (SD 1.6) to 3.8 (SD 1.2) (*p* < 0.001). In those initially receiving NTP who had TPM added, mean frequency dropped from 8.1 (SD 1.5) to 3.1 (SD 1.6), *p* < 0.001. Nonetheless, those randomized to have placebo added to their monotherapy also had a significant drop in their headache frequency, from 8.0 (SD 1.4) to 4.5 (SD 1.8) (*p* < 0.001).

**Fig. 2:**
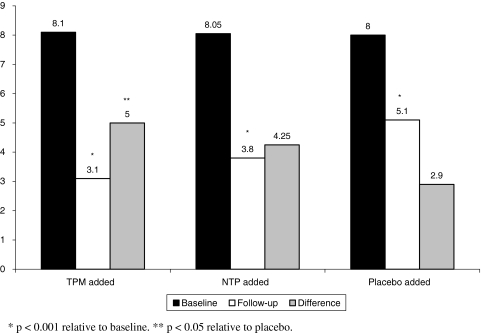
Mean number of monthly headache days at baseline and follow-up as a function of treatment groups

When comparing the reduction in headache frequency across all three groups, those randomized to receive TPM had a significantly increased reduction in headache frequency relative to those in placebo (mean 5.0 vs. 3.2, *p* = 0.02). Those receiving NTP had a numerical but not significant reduction relative to placebo. Difference in TPM and NTP were not significant. Data are summarized in Fig. 2.

**Fig. 3:**
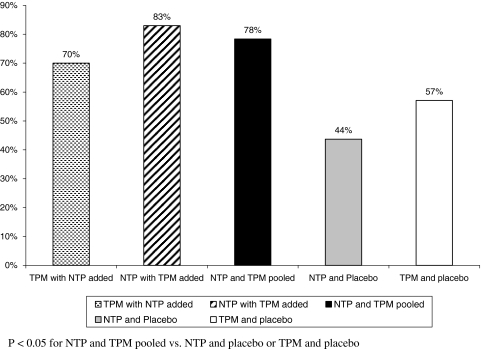
Proportion of individuals presenting at least 50% reduction in headache frequency as a function of treatment group

Of patients initially treated with TPM who had added NTP, 70% had at least 50% reduction in headache frequency; for those who added TPM, 83.3% of them achieved this substantial reduction. Together, 78.3% of patients randomized to receive the combination of the two drugs had at least 50% of headache reduction. For TPM + placebo users, 47% had at least 50% decreasing in headache frequency. For NTP + placebo users, 37% had at least 50% reduction in headache frequency. Overall, differences between switching to combination and continuing in monotherapy were significant (*p* = 0.04) (Fig. 3). The use of rescue medications (RD), although not used as an outcome comparator between the use of single drugs and the combination was clearly restricted to twice a week and did not reveal differences between groups. The average consumption of RD decreased from 10.2 to 4.3 days per month in combination group, from 10.7 to 4.5 in the TPM group and from 9.6 to 4.6 in the NTP group.

### Tolerability

Side effects among those who completed the treatment period were mild and consisted of dry mouth, paresthesia, weight loss, somnolence, weight gain, memory disturbances, hair loss and heartburn. Some patients presented two or three side effects simultaneously. Table 2 summarizes the incidence and characterization of the side effects presented by patients of the three groups. However, data on the occurrence of side effects among three female patients of the NTP + placebo group and two female patients of the combination group were not found for analysis. There were six drop outs in the combination group and six in the single drug groups (3 for each treatment regimen). The patients were lost to follow-up and reasons for dropping out were not identified (Fig. 1). Therefore, we decided to gather side effect profile information for those who completed the treatment period as presented in Table 2.

**Table 2:** Tolerability and side effect profile

Groups	Adverse events (%)
TPM + NTP	
65.9%	Dry mouth 18.2%
	Weight loss 11.4%
	Paresthesia 13.6%
	Somnolence 9.1%
	Weight gain 6.8%
	Hair loss 2.3%
	Heartburn 2.3%
	Dry mouth 4.5%
	Memory disturbances 6.8%
	Paresthesia and weight loss 9.1%
	Paresthesia. weight loss and memory disturbances 6.8%
TPM + placebo	
41.2%	Weight loss 17.6%
	Paresthesia 11.8%
	Weight gain 11.8%
NTP + Placebo	
36.8%	Weight gain 31.6%
	Hair loss 10.5%
	Weight gain and hair loss 5.3%

## Discussion

Managing the migraine patient is sometimes difficult, especially when they are referred from the neurologist to the headache specialist. Guidelines recommendations suggest that the goal of preventive treatment is to reduce headache frequency by at least 50%, based on the assumption that this reduction is likely clinically meaningful [13–15].

When a patient fails to respond as expected to appropriate therapy, or announces at the first consultation that he or she has already tried everything and nothing will work, it is important to identify the reason or reasons that treatment has failed. Among the several potential reasons, inadequate pharmacotherapy is listed [16]. Inadequate pharmacotherapy may occur if inappropriate treatments are selected, if excessive initial doses are used, if final doses are inadequate, if the duration of treatment is too short, if combination treatment is required, if the patient fails to absorb the drug, or if the patient is noncompliant [16]. Accordingly, although monotherapy is usually recommended, rational combination therapy is sometimes necessary.

Herein we found patients that were properly diagnosed and educated, and that had incomplete relief (measured by less than 50% reduction in headache frequency and subjective assessment of poor response) with adequate doses of first line medications, presenting significant improvement after being randomized to combination therapy, relative to the continuation on monotherapy (placebo added). Tolerability was not an issue for most patients.

Strengths of this study include the blinded design (to the best of our knowledge this is the first study to test combination vs. monotherapy of preventive medications in a blinded fashion), similar to what has been used for acute medications [16–20], as well as the use of medications that are considered to be first line for migraine, and are available as generics in several countries. In other words, we tried to be at the same time rigorous, while mimicking a “real-life” situation.

This study has important limitations as well. First, the sample size is small and did not allow multivariate comparisons or definitive conclusions whether one combination regimen is better than the other (starting with TPM and adding NTP or vice versa). More important, efficacy was assessed in a single time point, and not monthly for at least 3 months, as recommended by the IHS. Additionally, we used doses that are on the lower side of what is recommended for migraine prevention, i.e., 100–200 mg/day of TPM and 50–150 mg/day of a tricyclic antidepressant. The doses that we used could certainly be raised, either in mono or in polytherapy. Nonetheless, we have previously reported that tolerability is improved when combination therapy is done in the context of lower doses of medication, without apparent compromise of efficacy [21, 22]. One might even argue of whether the differences between monotherapy versus polytherapy could have been artificially inflated due to the superiority of TPM compared to NTP as a preventive drug. In addition, higher doses of NTP would have been more reasonable in terms of outcome instead of adding TPM.

However, we elected to use the combination of TPM and NTP based on the synergistic mechanism of actions and due to the fact that, in Brazil, the concerns of gaining weight are a daily strongly limiting factor in using usually recommended doses of NTP, which are higher than 30 mg/day. It is assumed that tricyclic antidepressants address the serotonergic and noradrenergic systems, while topiramate also modulates the glutamatergic and gabaergic systems. Accordingly, the combination of both may provide better efficacy due to the action on various possible neurotransmitter dysfunctions as suggested in migraine pathophysiology [22].

Our results are expected and supported by limited evidence. Pascual et al. tested the combination of TPM and different beta-blockers in patients who did not respond to the single use of medications [23]. Although the study was not blinded, they found that 62% of patients had at lest 50% reduction in headache frequency. The authors suggested that the combination could be useful due to the multi target action of the two drugs combined.

Barriers for migraine adequate care are several and important [24–26]. With the improvement in recognition and diagnosis, as well as better access to adequate acute medications, recent attention has been given to barriers of proper preventive medication use [11]. A sizeable proportion of migraineurs receiving preventive monotherapy are incompletely satisfied with the efficacy. Herein we present evidence that adding a second compound is associated with significant improve in headache frequency.
